# Contrast-Dependence of Temporal Frequency Tuning in Mouse V1

**DOI:** 10.3389/fnins.2020.00868

**Published:** 2020-08-25

**Authors:** Daniela Camillo, Mehran Ahmadlou, J. Alexander Heimel

**Affiliations:** Cortical Structure and Function Group, Netherlands Institute for Neuroscience, Institute of the Royal Academy of Arts and Sciences, Amsterdam, Netherlands

**Keywords:** V1, contrast, temporal frequency, divisive normalization, mouse, visual cortex

## Abstract

The perception of speed is influenced by visual contrast. In primary visual cortex (V1), an early stage in the visual perception pathway, the neural tuning to speed is directly related to the neural tuning to temporal frequency of stimulus changes. The influence of contrast on speed perception can be caused by the joint dependency of neural responses in V1 on temporal frequency and contrast. Here, we investigated how tuning to contrast and temporal frequency in V1 of anesthetized mice are related. We found that temporal frequency tuning is contrast-dependent. V1 was more responsive at lower temporal frequencies than the dLGN, consistent with previous work at high contrast. The temporal frequency tuning moves toward higher temporal frequencies with increasing contrast. The low half-maximum temporal frequency does not change with contrast. The Heeger divisive normalization equation provides a good fit to many response characteristics in V1, but does not fit the dependency of temporal frequency and contrast with set of parameters for all temporal frequencies. Different mechanisms for normalization in the visual cortex may predict different relationships between temporal frequency and contrast non-linearity. Our data could help to make a model selection.

## Introduction

While the signals that are produced by an image and leave the retina are dependent on the overall level of contrast, the interpretation of an image is largely independent of the overall contrast (Avidan et al., 2002). Reducing the contrast makes an image harder to see, but does not change its interpretation. Although we have some insight on how this independence of contrast arises by thresholding, we have no detailed understanding of this process even at the first stage of cortical visual processing. In the primary visual cortex (V1), neurons are responsive to local differences in image contrast, edges in particular (Hubel and Wiesel, 1959). In a good approximation, V1 neurons operate as spatiotemporal filters of the image contrast. Most investigations have focused on the interaction of spatial frequency filtering and contrast of grating stimuli. Initially, responses of V1 neurons were thought to be separable for contrast and spatial frequency, meaning that responses are the product of a function depending on stimulus contrast and a function depending on the spatial frequency (Albrecht and Hamilton, 1982). Later, it became clear that spatial frequency tuning and contrast are not completely inseparable in V1 in cat (Skottun et al., 1986), monkey (Sceniak et al., 2002; Priebe et al., 2006), and mouse (Heimel et al., 2010).

Likewise, the temporal frequency tuning and the contrast response of neurons in early visual cortical areas were first considered to be independent (Foster et al., 1985), but later found to depend on each other in macaque, cat and ferret V1 (Albrecht, 1995; Alitto and Usrey, 2004; Priebe et al., 2006) and macaque MT (Krekelberg et al., 2006; Pawar et al., 2019). In V1, temporal frequency tuning and speed are directly linked, because spatial and temporal frequency dependencies are separable in most of the individual neuronal responses (Tolhurst and Movshon, 1975). The interdependency of temporal frequency and contrast could thus underlie the so-called Thompson effect (Thompson, 1982; Thompson and Stone, 1997) that our perception of speed and temporal frequency is different at low contrast (Krekelberg et al., 2006). We wanted to understand the nature of the interaction of contrast and temporal frequency, and were interested to learn if this interaction is universal across mammals. In the mouse, V1 temporal frequency tuning has been measured at high contrast (Niell and Stryker, 2008; Gao et al., 2010; Durand et al., 2016), but the relationship between contrast and temporal frequency on responses has not been studied yet. Due to their small eye size, mice have about 100 times lower spatial acuity than humans (0.5 vs 60 cycles per degree; Prusky et al., 2000), but their temporal frequency tuning is more similar. In photopic conditions, contrast sensitivity in mice peaks at 1.5 Hz, six fold below humans (Burr and Ross, 1982; Umino et al., 2008), and mouse psychophysics of temporal contrast shares fundamental properties with human psychophysics (Umino et al., 2018).

We studied the responses in V1 of anesthetized mice to gratings of different temporal frequencies and contrasts. We found that responses do not factorize in contrast and temporal frequency dependencies, and that temporal frequency tuning moves to higher frequencies with higher contrast. V1 responses to many stimuli can be fitted by a divisive normalization model (Albrecht and Geisler, 1991; Heeger, 1992; Carandini and Heeger, 2011). Divisive normalization also describes the interdependency of contrast and spatial frequency of grating responses (Heimel et al., 2010). We investigated if divisive normalization also explains the relationship between contrast and temporal frequency in the responses, and if normalization operates equally across temporal frequencies. We found that, while the normalization model with a single saturation constant and exponent can approximately match V1 population responses for all combinations of temporal frequency and contrast, it does not describe the change in temporal frequency tuning with contrast for low and intermediate temporal frequencies.

## Materials and Methods

### Animals

We used male, 2–4 month old, calb2-cre mice bred on a C57BL6/J background (Strain #010774, Jackson laboratory), which we also used for investigating calretinin-positive cortical interneurons (Camillo et al., 2018). All animals were kept in a 12 h day/night cycle with access to food and water *ad libitum*. The experiments were carried out during the day cycle. All experiments were approved by the animal care and use committee of the Royal Netherlands Academy of Arts and Sciences. The experiments were performed in accordance with relevant guidelines and regulations.

### Extracellular Electrophysiology

Mice were injected with urethane (1.2 g per kg of mouse body weight, intraperitoneally) and chlorprothixene (8 mg per kg, subcutaneous). We injected atropine sulfate (0.1 mg per kg) to reduce mucous secretions. We maintained body temperature at 36.5–37°C with a heating pad and rectal probe. Additional doses of urethane were injected when a toe-pinch response was observed. The head was fixated with ear bars and a bite bar. During surgery, the eyes were protected from light by black stickers and from drying by Cavasan eye ointment. The scalp above visual cortex was removed and a very small craniotomy was made around 2,900–3,000 μm lateral and 300–500 μm anterior to Lambda. Laminar silicon electrodes (A1 × 16–5 mm-50–177-A16, 16 channels spaced 50 μm apart, Neuronexus) for extracellular recordings were inserted in the binocular region of V1. The signals were digitized at 24 kHz and band pass filtered between 0.5 and 10 kHz using a Tucker-Davis Technologies RX5 Pentusa. Signals were thresholded at 3× standard deviation to isolate spikes, and spikes were sorted after a principal component extraction by KlustaKwik (Harris et al., 2000) and custom-written Matlab (Mathworks) scripts.

### Visual Stimuli

Stimuli were back projected by a gamma-corrected Plus U2-X1130 85 Hz DLP projector onto a screen (Macada Innovision) placed 18 cm in front of the animal. Full screen size was 60 × 42 cm. Stimuli were produced by scripts using Psychophysics Toolbox 3 (Kleiner et al., 2007) running on Matlab. We first mapped the receptive fields of the units at the recording sites by presenting a 5 min movie (5 frames per second) of small white squares (approximately 5 degrees wide) in random positions on black background (ratio of white to black area: 1:30) (Ahmadlou et al., 2018). These receptive field positions were used to ascertain that we were recording in binocular V1. The next visual stimuli were full-screen, sine-wave, drifting gratings of 0.05 cycles per degree. Drift frequencies were 0.5, 1, 2, 4, 8, and 16 Hz. This corresponded to speeds of 10, 20, 40, 80, 160 and 320 degree per second. Grating contrasts were 10, 30, 50, 70, and 90%. In each 2 s long stimulus presentation, a grating was drifting in one of the eight cardinal and oblique directions. The stimuli were shown in pseudorandom order (i.e., shuffled per block). Each combination of contrast, temporal frequency and direction was shown five times for each recording. The screen was an equiluminant gray (10 cd m^–2^) for 1.5 s between the stimuli.

### Data Analysis

Analysis was done using Matlab scripts. For all stimuli, we computed the evoked visual responses, averaged over the duration of the stimulus, minus the spontaneous rate. The spontaneous rate was defined as the mean rate in the last 0.5 s before stimulus onset. We averaged the response for each combination of contrast and temporal frequency over all drift directions. Only units were included that had a minimum response (i.e., the evoked responses minus the spontaneous rate) of 1 spikes per second for at least one combination of temporal frequency and contrast. The response dependence on the temporal frequency was fitted with a difference of Gaussians (d.o.G.), i.e., *R*(*f*) = *R*_e_ exp(*−1/2 f^2^/w_e_^2^*) − *R*_i_ exp(−1/2 *f*^2^*/w_i_*^2^), where *f* is the temporal frequency, *R*_e_ and *w*_e_ are the gain and width of the positive Gaussian and *R*_i_ and *w*_i_ of the wider negative Gaussian. The fits were made by minimizing the summed squared error of the fit to the mean responses for all temporal frequencies, using the Matlab fminsearch implementation of the Nelder-Mead simplex algorithm. The fit was rejected if the optimal fit was found for *R*_e_ < 10^–4^ or *R*_i_ < 10^–4^ or *w_e_* > 10 × 16 Hz. The d.o.G. fit was used to calculate the optimal temporal frequency and the low and high half-maximum temporal frequencies at which the responses were half of the interpolated maximum response.

The response dependence on the Michelson contrast of the stimulus was fitted with a Naka-Rushton function, i.e., *R*(*c*) = *R_m_ c^*n*^*/(σ*^*n*^* + *c*^*n*^), where *c* is the contrast and *R*_m_, σ, and *n* are fitting parameters (Albrecht and Hamilton, 1982). The fits were made by minimizing the summed squared error of the fit to all responses for all contrasts plus very small contributions of σ^2^ and (*n* − 2)^2^ to reduce the degeneracy in fitting sometimes nearly linear data, using Matlab fminsearch. From the fit, the C50 value was interpolated as the contrast at which the response would be half of the response at 100% contrast.

The explained variance per unit for the temporal frequency and contrast response fits were calculated as 1 – {Σ*_i_* (*F*(*i*) − *R*(*i*))^2^}/{Σ*_i_* (*R*(*i*) − *R*_m_)^2^}, where *F*(*i*) are the fitted values for each frequency or contrast *i*, and *R*_m_ is the mean of all *R*(*i*)’s.

The population models in Section “Divisive Normalization” were fit by minimizing the norm of the difference between all the measured values and the fit values over all parameters, using Matlab fminsearch. For the normalization model, the optimal parameters were σ = 6.9 and *n* = 0.87. For the shunting-extended model, the optimal parameters were σ = 0.5, *τ* = 0.11/Hz, and *n* = 0.99.

An approximation for the LGN population tuning in the mouse was made by taking the values reported in the literature for the optimal temporal frequency, and low and high half-maximum responses for the LGN population (Tang et al., 2016), respectively, 3.2, 1.5, and 6.0 Hz and fitting a d.o.G. function with the same values (there were only band pass cells in the LGN). The fit was made by a stochastic search for the d.o.G. parameters that minimized the difference between the optimal, low half-maximum and high-maximum values of the d.o.G. with the literature values.

### Experimental Design and Statistics

The number of mice used for this study was determined before its start and was based on previous experience with determining feature tuning with mouse extracellular electrophysiology. The mice were randomly selected from the breeding stock. We used the Shapiro–Wilk test to test the C50 values and the optimal, low and high half-maximum values for normality. Most of these populations were not normally distributed at the 95% significance level and therefore we used non-parametric statistics for comparison and use the median and the bootstrapped standard deviation of the median as its standard error to describe the data. For comparisons of multiple populations, we used the Kruskal–Wallis test. For paired comparisons of two measurements of one population, we used the Wilcoxon signed-rank test. For comparing the fraction of units in two categories, we used the chi-square test. For testing a non-zero slope, we used the Matlab fitlm function, which applies a linear regression and computes the *p*-value for the *t*-statistic of the hypothesis test that the corresponding coefficient is equal to zero or not.

### Software Accessibility

The scripts for visual stimulus display and analysis of the data are available online at https://github.com/heimel/InVivoTools.

## Results

**FIGURE 1:**
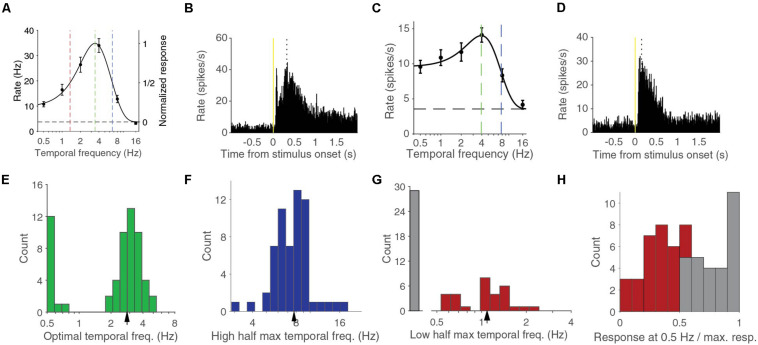
Temporal frequency response at 90% contrast can be band pass or low pass. **(A)** Temporal frequency tuning of an example band pass neuron. Error bars indicate s.e.m. around the mean rate during the stimulus presentation. Black dashed line indicates the spontaneous firing rate. Curve is a difference-of-Gaussian (d.o.G.) fit to the data. Green dashed line indicates the optimal temporal frequency at which the neuron is predicted to give most response. Red line indicates the lower half-maximum response frequency. Blue line indicates the higher half-maximum response frequency. **(B)** Peristimulus time histogram (PSTH) of the activity for all stimuli of the same neuron shown in **(A)**. Dotted line indicates the peak response time. **(C)** Temporal frequency tuning of an example low pass neuron. **(D)** PSTH for the same neuron as **(C)**. **(E)** Histogram of optimal temporal frequencies from d.o.G. fits. Arrowhead indicates median. **(F)** Histogram of high half-maximum temporal frequencies. Arrowhead indicates median. **(G)** Histogram of low pass cells and the lower half-maximum temporal frequencies of band pass cells. Arrowhead indicates the median of the band pass cells. **(H)** Histogram of the response at 0.5 Hz over the response at the optimal temporal frequency. In gray the low-pass cells, in red the band-pass cells.

We measured the response of neurons in V1 of anesthetized mice using linear silicon electrodes to drifting full-screen gratings of different contrasts and temporal frequencies (Figures 1A–D). For this report, we studied the 59 units in four mice that had a response larger than 1 spikes per second for at least one combination of contrast and temporal frequency. We first analyzed the temporal frequency dependence at 90% contrast, the highest stimulus contrast that we used, and reproduced previous findings (Niell and Stryker, 2008; Gao et al., 2010; Durand et al., 2016). The temporal frequency tuning of these cells could be well-fitted with a difference-of-Gaussian (d.o.G.) curve (median explained variance was 97%, examples in Figures 1A,C). The median optimal temporal frequency was 2.83 ± 0.14 Hz (bootstrapped standard deviation) (Figure 1E). The median high half-maximum temporal frequency was 7.7 ± 0.3 Hz (Figure 1F). Units were termed band-pass cells if they responded to stimuli shown at 0.5 Hz, the lowest temporal frequency that we tested, at less than half the interpolated response to the optimal temporal frequency. About half, 30 of 59 (51%) of the units were band-pass cells. These band-pass cells had a median low half-maximum temporal frequency of 1.09 ± 0.07 Hz (Figure 1G). The other half were considered low-pass cells, although the histogram of the ratio of the response at 0.5 Hz over the response at the optimal temporal frequency shows that there is no strict division between low-pass and band-pass cells (Figure 1H).

### Varying Temporal Frequency and Contrast

**FIGURE 2:**
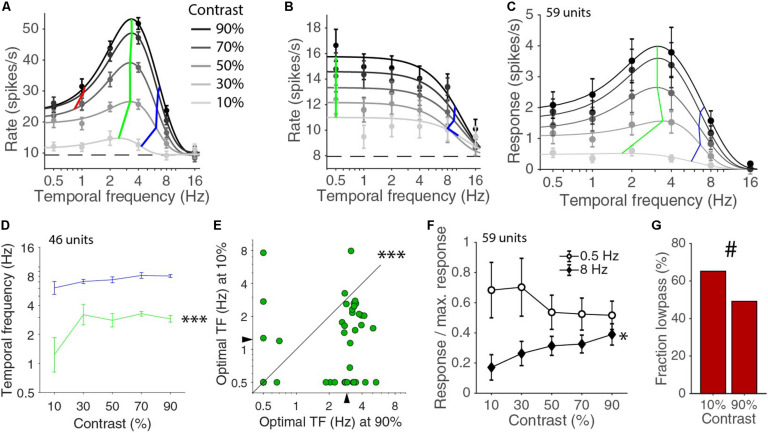
Relationship of temporal frequency and contrast. **(A)** Temporal frequency tuning for different contrasts for an example band pass neuron. Error bars indicate s.e.m. around the mean rate during the stimulus presentation. Dashed line indicates the spontaneous rate. Black curves are d.o.G. fits, independently made for each contrast. Green lines connect the optimal temporal frequencies for each contrast. Blue lines connect high half-maximum responses. Red lines connect low half-maximum response when they exist. **(B)** Example like **(A)** for a low pass neuron. **(C)** Temporal frequency tuning for the mean population response for all units. Green and blue lines are connecting the optimal and high half-maximum responses for the fits of the population averages. **(D)** Median optimal (green) and high half-maximum temporal frequency (blue) for different contrasts for all individual units. Error bars are bootstrapped s.e.m. There is a difference across contrasts for optimal frequency (^∗∗∗^*p* = 0.00004, Kruskal–Wallis test, 46 units in four mice), but only a trend for a change in the high half-maximum (*p* = 0.10, Kruskal–Wallis test). **(E)** Optimal temporal frequency is lower at 10% contrast than at 90% contrast (^∗∗∗^*p* = 0.00012, Wilcoxon signed-rank). **(F)** Response at 0.5 and 8 Hz normalized by the maximum response at each contrast. The relative response at 8 Hz increases with contrast (^∗^*p* = 0.029, non-zero slope test). **(G)** Fraction of low pass cells at 10 and 90% contrast (#, 29/59 at 90% contrast, 30/46 at 10%, *p* = 0.10, chi-square test).

Next, we considered the temporal frequency tuning across all the presented contrasts of 10, 30, 50, 70, and 90%. For 46 of the 59 units, the temporal frequency tuning could be well fitted with a d.o.G. for all contrasts. The median explained variances were 97, 97, 96, 91, and 82% for contrasts of 90, 70, 50, 30, and 10%, respectively. For the other 13 cells, the fit was too poor, because the response at the 10% was completely absent or so low that the number of repetitions that we used was insufficient to provide an accurate measurement of the response. Cells that were band-pass in temporal frequency responses for high contrasts often became low-pass at lower contrasts (Figure 2A). Low-pass cells at high contrasts were always also low-pass at low contrasts (Figure 2B). The mean temporal frequency responses for the whole population were also well described by different d.o.G. functions for the different contrasts (Figure 2C). The shape of the curve and the optimal temporal frequency and high half-maximum temporal frequencies of the population response were fairly constant above 30% contrast except for a gain change. Looking at the values of individually fitted temporal tuning curves, we noticed that there was significant difference in the optimal temporal frequency across contrasts (*p* = 0.00004, Kruskal–Wallis test, d.f. = 225, χ^2^ = 25.6, 46 units in four mice; Figure 2D). A similar, but not significant, trend was also present in the high half-maximum temporal frequency across contrasts (*p* = 0.10, Kruskal–Wallis test, d.f. = 225, χ^2^ = 7.7; Figure 2D). More specifically, the optimal temporal frequency at 10% was much lower than the optimal temporal frequency at 90% (median ± s.e.m. at 10% contrast: 1.23 ± 0.40 Hz; at 90% contrast: 2.90 ± 0.19 Hz; *p* = 0.00012 Wilcoxon signed-rank, *z* = 3.8, 46 units in four mice; Figure 2E). The high half-maximum temporal frequency was also significantly lower at 10% contrast than it was at 90% contrast (median ± s.e.m. at 10% contrast: 6.08 ± 0.99 Hz; at 90% contrast: 8.07 ± 0.27 Hz; *p* = 0.033 Wilcoxon signed-rank, *z* = 2.1). The response at 8 Hz relative to the maximum response increased with increasing contrast (*p* = 0.029, non-zero slope test, *F* = 4.9; Figure 2F). This did not happen at 0.5 Hz. If anything, there was a drop of relative response with increasing contrast and the slopes of the 0.5 and 8 Hz curve were significantly different (*p* = 0.0017, non-zero slope test on difference, *F* = 9.99; Figure 2F). The low half-maximum temporal frequency was smaller at low contrast (at 10% contrast: 0.89 ± 0.11 Hz; at 90% contrast: 1.14 ± 0.09 Hz), but this was just a trend (*p* = 0.10, Wilcoxon signed-rank, statistic = 79). At 10% contrast, there were only 16 band-pass neurons out of the 46 fitted by a d.o.G. (low-pass were 29 of 59 units at 90% contrast, 30 of 46 at 10% contrast, *p* = 0.10, chi-square test; Figure 2G). Overall, the temporal frequency tuning in V1 shifts toward higher temporal frequencies with increasing contrast.

**FIGURE 3:**
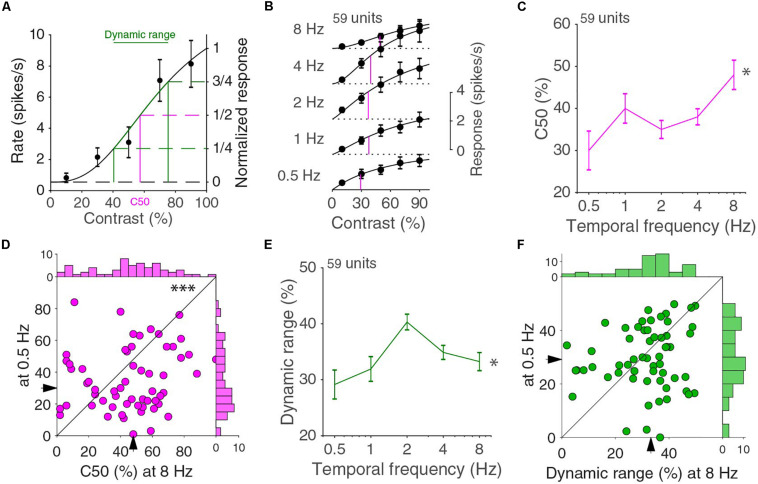
Contrast response function for different temporal frequencies. **(A)** Contrast response function for an example neuron. Error bars indicate s.e.m. around the mean. The curve is a fit with a Naka-Rushton function. C50 is the contrast at which the fitted response is half the response at 100% contrast. The dynamic range is the difference in contrasts that evoke one and three quarters of the 100% contrast response **(B)**. Population averaged contrast responses across temporal frequencies. In magenta, the C50s of the Naka-Rushton fits of the population responses are indicated. Independent fits are made for all curves. **(C)** Median C50 varies across temporal frequencies (^∗^*p* = 0.016, Kruskal–Wallis test, 59 units in four mice). Error bars indicate bootstrapped s.e.m. **(D)** C50 at 0.5 and 8 Hz (^∗∗∗^*p* = 0.0057 Wilcoxon signed-rank). Histograms are shown on top and to the right. **(E)** The dynamic range differs across temporal frequencies (^∗^*p* = 0.011, Kruskal–Wallis test). **(F)** Dynamic range at 0.5 and 8 Hz (*p* = 0.51, Wilcoxon signed-rank). Histograms are shown on top and to the right.

Other than looking at how the temporal frequency tuning changed with contrast, we can also look at how the contrast response function changed with temporal frequency. The contrast dependence at a single temporal frequency can be fitted by a Naka-Rushton function (Albrecht and Hamilton, 1982). For higher contrasts, the Naka-Rushton curves have a decreasing steepness, referred to as saturation (Figure 3A). Occasionally, neurons were “super-saturated” and response decreased for the highest contrasts, but generally there was a very good fit (median explained variance was 98%). The C50 value, i.e., the interpolated contrast at which the cell responds at half the extrapolated 100% contrast response, is a common way to give an indication of the range of contrast where a cell is most sensitive. A high or low C50 indicates that the cell is most sensitive to, respectively, high or low contrasts. The population response curves showed differences in C50 across temporal frequencies (Figure 3B). The population contrast curves of Figure 3B, however, are more linear than the individual tuning curves and do not necessarily accurately reflect the changes in the individual neuronal contrast response curves. The medians of the individual units C50, however, showed similar differences across temporal frequencies (*p* = 0.016, 59 units in four mice, Kruskal–Wallis test, d.f. = 294, χ^2^ = 12.1, Figure 3C) and the C50 at 8 Hz is higher than at 0.5 Hz (*p* = 0.0057, Wilcoxon signed-rank, *z* = 2.8; Figure 3D). This again illustrates that response functions did not factorize in separate contrast and temporal frequency dependent functions. The C50 value alone does not completely describe the contrast response function. In particular, it does not capture whether the response changes over the full range of contrasts or only over a narrow range. For this reason, we also computed the dynamic range, i.e., the difference between the contrasts that evoke one quarter and three quarters of the maximum response (Figure 3A). A cell with a C50 of 50% contrast with a response that grows linearly with contrast has a dynamic range of 50%. A cell that has a very steep increase in response around the C50 contrast has a much lower dynamic range. The median dynamic range had a dependence on temporal frequency (*p* = 0.011, Kruskal–Wallis test, d.f. = 294, χ^2^ = 13.0; Figure 3E), and peaked at 2 Hz. The distributions of the dynamic range at 0.5 and 8 Hz, however, were not different from each other (*p* = 0.51, Wilcoxon signed-rank, *z* = 0.66; Figure 3F).

### Divisive Normalization

**FIGURE 4:**
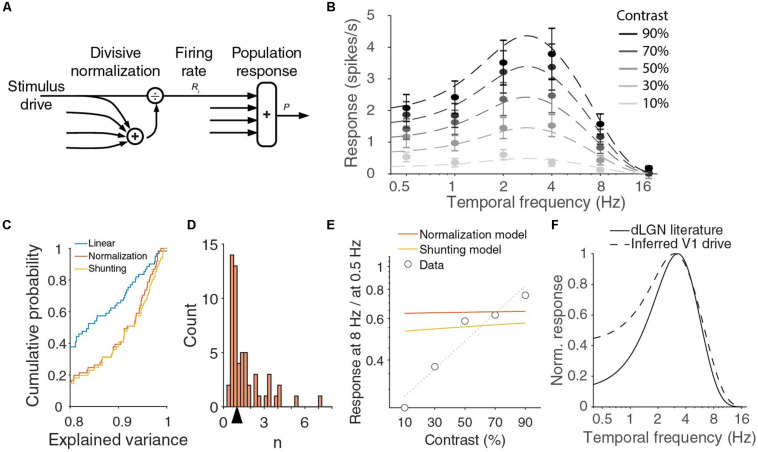
V1 output can be fit with normalization model, but inferred drive is lower pass than dLGN output. **(A)** In the normalization model, the response of a neuron is described by a stimulus drive divided by the sum of a pool of such inputs. **(B)** Population responses across temporal frequencies and contrasts can be fit with divisive normalization of d.o.G. temporal frequency tuning of the input. Red curve is a fit with *R*_m_ = 37 spikes/s, σ = 6.9, *n* = 0.87, *R*_e_ = 1 spikes/s, *w*_e_ = 4.7 Hz, *R*_i_ = 0.66 spikes/s, *w*_i_ = 1.5 Hz. Error bars denote s.e.m. around mean. **(C)** Cumulative histogram of the explained variances when all individual units are fit with a linear model, the normalization model, or the shunting-extension of the normalization model. **(D)** The histogram of n for fitted to all individual units. The arrow indicates the median 0.99. **(E)** The ratio of the response at 8 Hz over the response at 0.5 Hz strongly varies with contrast in the data, but this is not true in the optimal fit of the normalization model or for the shunting model. Dotted line is linear fit to the data. **(F)** Full contrast dLGN output from literature (Tang et al., 2016) (solid line) does not match the stimulus drive from the normalization equation (dashed line).

Our results thus clearly show that the mouse V1 population response is not a product of a function dependent on the temporal frequency and a function dependent on the stimulus contrast. The same is the case for the combination of spatial frequency and contrast (Heimel et al., 2010). The interdependence of response on contrast and spatial frequency was accurately described by divisive normalization. Divisive normalization is characterized by the normalization equation, describing the response *R*_i_ of neuron *i* by:

$$R_{i}=D_{i}^{n}\,/\,\left(\sigma^{n}+\sum_{k}D_{k}^{n}\right)\;\left(1\right)$$

where the enumerator *D*_i_ describes the driving input into the neuron and the denominator is a saturation constant σ plus the sum of a large number of driving inputs *D*_k_, the normalization pool (Figure 4A; Heeger, 1992; Carandini and Heeger, 2011). The exponent *n* is a parameter signifying the rectification stage of the model. If we consider the population response *P* = ∑_*i*_*R*_*i*_, we find *$P=\sum_{i}D_{i}^{n}\,/\,\left(\sigma^{n}+\sum_{k}D_{k}^{n}\right)$*. We have established experimentally that the population response does not factorize, but let us assume the driving inputs into V1 approximately do, i.e., there exist functions *d*_i_(*f*) such that *D_i_* = *c d_i_*(*f*)^1/^*^*n*^* with contrast *c* and temporal frequency *f*. We can then define *T*(*f*) = Σ*_i_ d_i_*(*f*), and find

$$P\,\left(c,f\right)=c^{n}\,/\,\left(\sigma^{n}\,/\,T\,\left(f\right)+c^{n}\right)\;\left(2\right)$$

This is a Naka-Rushton function, just as those that were used to fit the contrast responses of individual neurons, but the shape of the function depends on the temporal frequency. From Eq. (2) follows that there should be a single temporal frequency tuning function *T*(*f*) and two parameters σ and *n* to fit the V1 population response at all contrasts and temporal frequencies. We find that indeed the 30 population responses (six temporal frequencies at five contrasts) can be well fitted by the normalization model with seven parameters (*n*, σ and the five d.o.G. parameters for T). An example fit (with T described by a difference-of-Gaussians) explaining 98% of the variance in the means is shown in Figure 4B, but there is a large range of parameter values with a similar goodness of fit. For all good fits, σ*^*n*^*/*T*(*f*) is much larger than 1 and *n* is close to 1. In those cases, the population response is approximately equal to *c T*(*f*)/σ. In fact, fitting the joint temporal frequency and contrast tuning with a function *c T*(*f*) that is just linear in contrast, also explained 97% of the variance, and is thus an equally good fit with two parameters less (*n*, σ). The contrast-temporal frequency curves of individual neurons, however, are much more poorly fit by fits that are linear in contrast, than by the normalizing model with *n* and σ optimized for each unit (median explained variance of linear model: 83%, normalization model: 92%; *p* = 10^–11^ Wilcoxon, *z* = −6.8; Figures 4C,D). The normalization model thus provides a much better, but still not perfect fit. If we look at the population fits in Figure 4B more closely, we see that they are relatively poor at low temporal frequencies. The normalization model explained only 92 and 94% of the variance at 0.5 and 1 Hz, while it explained 99, 99, and 97% at 2, 4, and 8 Hz. The fits undershoot for the lowest contrasts, and overshoot for the highest contrasts. Furthermore, while the data showed that the optimal temporal frequency shifts with contrast, Figure 4B shows that the optimal temporal frequency does not change with contrast. Indeed, taking the derivative with respect to *f* of the population response in Eq. (2), we find

$$\frac{d\,P\,\left(c,f\right)}{d\,f}=\frac{\sigma^{n}\,c^{n}}{{\left[\sigma^{n}+c^{n}\,T\,\left(f\right)\right]}^{2}}\,\frac{d\,T\,\left(f\right)}{d\,f}.$$

Therefore, the maximum of the population response *P* will be at the maximum of *T*(*f*) at *f* = *f*_opt_ where *dT*(*f*)/*df* = 0, independently of the contrast. Furthermore, taking the derivative of *P*(*c,f*)/*P*(*c,f*_opt_) with respect to contrast, we find

$$\frac{d}{d\,c}\,\frac{P\,\left(c,f\right)}{P\,\left(c,f_{o\,p\,t}\right)}=\frac{n\,c^{n-1}\,\sigma^{n}\,\left\{T\,\left(f_{o\,p\,t}\right)-T\,\left(f\right)\right\}}{T\,\left(f_{o\,p\,t}\right)\,T\,\left(f\right)\,{\left\{\frac{\sigma^{n}}{T\,\left(f\right)}+c^{n}\right\}}^{2}}>0,$$

because *T*(*f*_opt_) > *T*(*f*). This means that the response at a suboptimal temporal frequency will grow relatively faster with contrast than the response at the optimal frequency *f*_opt_. Thus the responses at 0.5 and 8 Hz, divided by the maximum response, should grow with contrast. In Figure 2F, we presented that this was true at 8 Hz, but not at 0.5 Hz. Indeed, if we plot the ratio of the response at 8 Hz and at 0.5 Hz, we see that the experimental data show a strong increase with contrast, while the optimal fit of the normalization model predicts the ratio to be almost independent of the stimulus contrast (Figure 4E).

One assumption implicitly made in the normalization Eq. (1) is that there is a single pair of saturation constant σ and exponent *n* for different stimuli. Any neural implementation of normalization, however, will be dynamic and there could be an interaction of the stimulus dynamics and normalization. Indeed, one suggested implementation of divisive normalization by shunting inhibition (Carandini and Heeger, 1994; Carandini et al., 1997) predicts that σ grows with the temporal frequency of the stimulus. This would allow *P*(*c,f*)/*P*(*c,f*_opt_) to decrease with contrast for small frequencies (again can be shown by inserting Eq. (2) and taking the derivative), and potentially fit the decreasing relative response at 0.5 Hz (Figure 2F). However, if we substitute σ + *τ f* for σ, and optimize the fit for all data, we find essentially the same fit as for the model with fixed σ and do not get a better fit for the ratio of the responses to 8 and 0.5 Hz (Figure 4E, shunting model). If we reconsider Eq. (2), however, we see that any frequency-dependency of σ can be absorbed by choosing a different *T*(*f*). This explains why we get almost the same fit. The reason for the small difference between the models in Figure 4E is because we constrained *T*(*f*) to be a difference-of-Gaussians. If we release this requirement, the shunting model and the regular model give exactly the same fits, with an explained variance of 98%. Fitting individual units with this shunting model slightly improves the fits, as it has an extra degree of freedom and includes the static normalization equation (*p* = 8 × 10^–6^, Wilcoxon, *z* = −4.4; Figure 4C), but the median explained variance is lower for the extended model when we adjust it for the extra model parameter (median adjusted explained variance normalization model: 0.89, shunting model: 0.88).

The normalization model predicts the temporal frequency tuning of the driving input to V1 to be equal to *T*(*f*)^1/^*^*n*^* (Figure 4F). It is difficult to measure the driving input into V1, but we expect it to be close to the signal leaving the dLGN. The temporal frequency dependence of the mouse dLGN has been measured with full contrast stimuli (Grubb and Thompson, 2003; Tang et al., 2016). From the reported optimal temporal frequencies (median 3.2 Hz) and low and high half-maximum frequencies (medians of 1.4 and 6.0 Hz, respectively), we can infer the dLGN population response (Figure 4F). There is a large difference between the prediction from the normalization model and the measured dLGN response at the lower temporal frequencies.

We conclude that our findings are consistent with divisive normalization operating at the higher temporal frequencies, but that either divisive normalization is not operating as described by Eq. (1) at the lower temporal frequencies, or that the dependencies on contrast and temporal frequency in the driving inputs into V1 do not factorize.

## Discussion

We found that the shape of contrast tuning curves of mouse V1 neurons depended on the temporal frequency of the stimulus and vice versa, the shape of the temporal frequency tuning curve depended on the stimulus contrast. We measured this with drifting gratings, as commonly done (Foster et al., 1985; Priebe et al., 2006; Gao et al., 2010). The gratings had a fixed spatial frequency, and changing temporal frequency was equivalent to changing the drifting speed. It has been established, however, that in V1 dependencies on spatial and temporal frequency are mostly separable (Tolhurst and Movshon, 1975), and rather than looking at speed tuning, it is natural to focus on spatial and temporal frequency tuning.

Our findings at 90% contrast are very comparable to previous measurements of temporal frequency tuning at 80–100% contrast in mouse V1 (Niell and Stryker, 2008; Gao et al., 2010; Durand et al., 2016). In close agreement to our finding that 49% of the cells were low-pass tuned, Gao et al. (2010) found that 44% were low-pass. The two early studies found a peak response for temporal frequencies between 1 and 2 Hz. This is slightly below our median peak frequency (2.83 Hz), which exactly matches the 2.8 Hz found by Durand et al. (2016). We cannot give a reason for this difference. Perhaps there was a difference in the depth of anesthesia across the studies, although there was no significant difference in peak frequency between the awake and anesthetized condition (2.8 vs 2.99 Hz; Durand et al., 2016). Niell and Stryker (2008) used counter-phase changing gratings, different from our drifting gratings, but Gao et al. (2010) used drifting gratings. At low luminance, temporal frequency tuning is strongly dependent on luminance (Ferry-Porter law; Bex and Langley, 2007; Umino et al., 2008), but the previous studies and ours were done at similar, photopic, light levels, where tuning is independent of luminance. Our median optimal temporal frequency is below the 4 Hz found for rats (Girman et al., 1999), but in line with other nocturnal and crepuscular animals that are strongly dependent on rod vision (Heimel et al., 2005).

The peak frequency in V1 is below the peak frequency in the mouse dLGN (3–4 Hz; Grubb and Thompson, 2003; Durand et al., 2016; Tang et al., 2016). Furthermore, while half of the cells in mouse V1 are low-pass for temporal frequency, the cells in the dLGN are consistently band-pass (Grubb and Thompson, 2003). The temporal frequency tuning thus substantially moves toward lower frequencies in the mouse like it does in the monkey (Hawken et al., 1996). This shift has been hypothesized to be caused by a combination of intracortical inhibition and thalamocortical NMDA receptors (Krukowski and Miller, 2001).

We found that the temporal frequency tuning characteristics were dependent on contrast, in particular at low contrast. Responses at high temporal frequencies were relatively more reduced at lower contrasts than responses at more optimal temporal frequencies (Figure 2F). Relative response at low temporal frequencies, on the other hand, were not more reduced at lower contrasts. Reducing contrast also slightly lowered the preferred temporal frequency (Figure 2D). In carnivores, the responses at low and high temporal frequencies also grew more with increasing contrast than responses at the optimal temporal frequency (Holub and Morton-Gibson, 1981). In particular, the responses at high temporal frequencies grow faster at higher contrasts, and thus also leading to a shift toward a higher temporal frequency preference with increasing contrast (Albrecht, 1995; Alitto and Usrey, 2004). This influence of contrast on temporal frequency tuning is different from its influence on spatial frequency tuning. In the macaque, the optimal spatial frequency is independent of contrast and both the low and high half-maximum frequencies move away from the optimal frequency with increasing contrast (Sceniak et al., 2002). In the mouse, the contrast dependence has not been measured down to the typical low half-maximum frequency (which requires very large stimuli due to the low visual acuity of mice), but the response to high spatial frequency gratings was also much more sensitive to contrast than the response to gratings of the optimal spatial frequency (Heimel et al., 2010). The effect of contrast on spatial frequency tuning follows from the divisive normalization behavior of visual cortex (Heimel et al., 2010). According to the normalization model, the response of a neuron is well described by dividing its driving input by a constant and the sum of the activity of a normalizing pool of inputs (Heeger, 1992; Carandini and Heeger, 2011). For spatial frequencies that evoke much response, the activity level of the normalizing pool is higher and the driving input is divided by a larger number than for spatial frequencies that evoke little response. The effect is thus a widening of the tuning curve with increasing contrast. At high temporal frequencies, the effect of contrast on the population response is as predicted by the normalization model (see Figure 2F). Higher temporal frequencies cause less activity in the normalization pool than more optimal temporary frequencies, resulting in less normalization and thus more dependence on contrast. At low temporal frequencies, the stationary normalization Eq. (1) predicts the same effect, but the data do not show this (Figure 2F). One of the assumptions implicitly made in Eq. (1) is that the saturation constant σ and the exponent *n* are independent of the stimulus. This assumption holds across orientations and spatial frequencies (Heeger, 1992). At the introduction of the normalization model, the validity of this assumption was not asserted for different temporal frequencies, but it was noted that the model could explain the contrast-dependent changes in temporal frequency tuning that were found in the cat (Holub and Morton-Gibson, 1981; Heeger, 1992; Albrecht, 1995). The stationary normalization equation, however, does not accurately describe the responses across temporal frequencies and contrasts with a single set of σ and *n* (Carandini et al., 1997). It could still be, however, that a single divisive normalization mechanism is operating, but that the interaction of a changing stimulus and the normalization mechanism leads to a different set of parameters in the equation describing the stationary state. Currently, we do not know how divisive normalization is implemented in the visual cortex. A number of mechanisms, such as (shunting) inhibition (Carandini and Heeger, 1994), excitation (Sato et al., 2016) and short-term synaptic depression (Carandini et al., 2002) have been proposed to underlie the normalization phenomenon in visual cortex, but there is not one mechanism that is consistent with all effects. Carandini et al. (1997) have shown how shunting inhibition could lead to divisive normalization with a saturation constant σ that grows with increasing stimulus frequency. The resulting stationary normalization equation, however, produces the same fit to the data as the model without this extra degree of freedom, if we also optimize the frequency-dependency of the driving input.

Even without knowing the mechanism underlying normalization, looking at normalization as a dynamic process offers a possible explanation for the relatively poor fit of the data at lower temporal frequencies. Consider that the normalization mechanism is operating on a time scale that is faster than that of the low temporal frequencies (0.5 Hz) that we used. The activity of many neurons in V1 is modulated by the stimulating temporal frequency. A simple cell would be very responsive during one period of the stimulus cycle. Perhaps the effects of normalization diminish quickly during the responsive period of a cell. For low temporal frequencies, normalization would thus have no effect during most of the responsive period of the cell and would not change the average firing rate as much as it would do for higher temporal frequencies.

One more assumption that was implicitly made in deriving the stationary normalization Eq. (1) was that the activity in the normalization pool is equal to the average of the population activity. Of course, this can only be an approximation, because the neurons certainly do not have instantaneous access to the population activity across the entire visual field (Reynaud et al., 2012). In our case, however, we have used full screen gratings and recorded from neurons that had their receptive field away from the screen borders. The stimulus input would thus at least have been relatively homogeneous for nearby neurons which have similar receptive fields. The normalization pool, however, could have a different temporal frequency tuning than the population activity. This extra freedom in the fit of the model would certainly produce a more accurate fit to the data. It could also be that the normalization pool has a local polarity (dark/light) preference, and therefore oscillate with the stimulus frequency. In this case, it also becomes necessary to estimate the time averaging by the normalization mechanism. To fit human steady state visual evoked potentials of masking stimuli with the normalization model, a temporal averaging window of 26 ms provided the best fit (Tsai et al., 2012). If one, however, allows the normalization pool and the population pool to vary independently for different stimulus parameters, the predictive power of the normalization model disappears.

An entirely different explanation is that normalization does operate also at lower temporal frequencies, but that the assumption that the contrast and temporal frequency dependencies of the input driving V1 factorize does not hold. We do not know the precise input into V1, but we do know that indeed in the macaque this assumption fails. Changes in temporal frequency altered the contrast tuning in dLGN (Derrington and Lennie, 1984; Dhruv et al., 2009). In the ferret, the difference in contrast gain at low and high temporal frequency was not higher in V1 than the difference already present in the LGN (Alitto and Usrey, 2004). Furthermore, even in the retina (of the cat) contrast and temporal frequency do not completely separate for low temporal frequencies (Shapley and Victor, 1978). Responses of X and Y retinal ganglion cells to low frequencies of modulation (<1 Hz) grew less than proportionally with contrast. Response amplitudes at higher modulation frequencies scaled approximately proportionally with contrast. The source of the interdependency of temporal frequency and contrast in mouse V1 responses may thus already lie in the LGN or the retina.

This explanation why the normalization model poorly fits the data, leaves open the possibility that divisive normalization is operating in the V1 exactly as predicted. However, along the visual hierarchy in human cortex, the temporal frequency tuning becomes progressively more low-pass (Mullen et al., 2010). This would not follow from a normalization model working the same at all temporal frequencies. As discussed previously, dynamic implementations of divisive normalization may lead to frequency dependence of the saturation constant (Carandini et al., 1997), but there could also be other mechanisms operating in the visual system to change temporal frequency tuning to lower frequencies. This may be the combination of intracortical inhibition and NMDA receptor signaling hypothesized to be responsible for the change in temporal frequency from dLGN to V1 (Krukowski and Miller, 2001). It will be interesting to understand if this would correctly predict our data on the interplay between temporal frequency and contrast in the responses. Furthermore, it may give a mechanism underlying the Thompson effect that contrast and perceived speed and flicker are not completely separated (Thompson, 1982; Thompson and Stone, 1997).

More than 50 years after the first psychophysical measurements of the dependence of contrast sensitivity on temporal frequency (Robson, 1966), we find that we still do not know how the limits set by the retina are changed into the limits of our perception. Our measurements could help to select between candidate neural implementations of the normalization model linking visual input to perception.

## Data Availability Statement

The raw data supporting the conclusions of this article will be made available by the authors on request, without undue reservation.

## Ethics Statement

The animal study was reviewed and approved by DEC and IVD KNAW.

## Author Contributions

DC and MA performed the experiments. DC, MA, and JH devised the experiments. JH wrote the manuscript. All authors contributed to manuscript revision, read, and approved the submitted version.

## Conflict of Interest

The authors declare that the research was conducted in the absence of any commercial or financial relationships that could be construed as a potential conflict of interest.
